# Management and Outcome of Parathyroid Carcinoma-Induced Primary Hyperparathyroidism: A Single-Centre Experience

**DOI:** 10.1155/2021/5397941

**Published:** 2021-10-07

**Authors:** Loredana De Pasquale, Antonio Mario Bulfamante, Giovanni Felisati, Luca Castellani, Giorgio Ghilardi, Alberto Maria Saibene

**Affiliations:** ^1^Thyroid and Parathyroid Surgery Service—Otolaryngology Unit, ASST Santi Paolo e Carlo, Department of Health Sciences, Università Degli Studi di Milano, Via Antonio di Rudinì 8, 20142 Milan, Italy; ^2^Otolaryngology Unit, ASST Santi Paolo e Carlo, Department of Health Sciences, Università Degli Studi di Milano, Via Antonio di Rudinì 8, 20142 Milan, Italy; ^3^Department of Health Sciences, Clinica Chirurgica Generale, Università Degli Studi di Milano, Via Antonio di Rudinì 8, 20142 Milan, Italy

## Abstract

**Background:**

Parathyroid carcinoma (PC) is the rarest endocrine cancer and an infrequent cause of primary hyperparathyroidism (PHPT), responsible for less than 1% of cases. Due to its rarity, treatment is challenging.

**Methods:**

A retrospective cohort study on 462 patients referred for parathyroidectomy to Thyroid and Parathyroid Unit at Santi Paolo e Carlo Hospital, Milan, Italy, from 2011 to 2021. We identified and individually described the patients affected with PC. Then, we split all patients treated for PHPT into four groups based on the cause: PC, adenoma, atypical adenoma, and hyperplasia. Patients' demographics, preoperative evaluation results, intraoperative findings, and outcomes for the PC group were compared with groups of PHPT due to benign causes.

**Results:**

Eight cases of PC were identified, five males and three females. Seven cases presented with symptoms of hypercalcemia and one with a neck mass. Five underwent en bloc resections and three local excisions. Histopathological features showed capsular invasion in four patients, capsular and soft tissue invasion in three patients, and vascular invasion in one case. No patients had distant metastasis. One patient was classed as high risk based on the Schulte classification system. All patients treated for PC were alive and disease-free at a mean follow-up of 38.4 months. When compared with other PHPT patients, PC patients were more frequently male and had higher preoperative blood calcium and PTH and lower phosphate levels, larger and heavier parathyroids excised, lower postoperative calcium, and a higher rate of postoperative hypoparathyroidism.

**Conclusion:**

Our study highlights some aspects valuable to suspect PC and differentiate PHPT-PC from benign causes of PHPT preoperatively. Preoperative suspicion of malignancy is essential to guarantee the best course of treatment for patients. Although limited for size and follow-up, the excellent outcome of our series seems to support the value of both surgery extension and risk class according to the Schulte classification as possible prognostic factors for recurrence.

## 1. Introduction

Parathyroid carcinoma (PC) is a rare malignancy, accounting for 0.005% of all cancers [1], the rarest endocrine cancer, as well as the rarest cause of primary hyperparathyroidism (PHPT). Less than 1% of PHPT cases are due to PC [2].

Most patients affected with this rare malignant tumour present with either sporadic primary hyperparathyroidism or primary hyperparathyroidism in the context of a genetic endocrine syndrome, namely, Multiple Endocrine Syndrome Type I (MEN I), Multiple Endocrine Syndrome Type II (MEN II), Hyperparathyroidism Jaw Tumour Syndrome (HPT/JT), and Familial Isolated Primary Hyperparathyroidism (FIHP) [3–5].

Only a few cases of PC have normal serum PTH levels [6–8].

Diagnosis is generally confirmed only after surgery by detailed pathological analysis unless there is preoperative evidence of gross local invasion, cervical lymph nodes, or distant metastases.

The surgical approach offers the best disease control rates, while there is no evidence supporting other PC management options.

Since this cancer is rare, a general consensus about the staging system and prognostic factors is lacking. Schulte proposed two types of classification based on histopathological criteria as good predictors of recurrence and survival [9].

This study aims to review our experience with 8 PC patients concerning diagnosis, treatment, and outcomes. In order to provide a more solid reference framework, we shall compare the demographics, preoperative status, complications, and postoperative status of these cases with those of a retrospective cohort of patients affected by primary hyperparathyroidism due to non-PC-related causes (typical and atypical parathyroid adenoma and parathyroid hyperplasia).

## 2. Materials and Methods

This retrospective cohort study is on 462 consecutive adult patients referred for parathyroidectomy to the Thyroid and Parathyroid Unit at Santi Paolo e Carlo Hospital, Milan, Italy, from 2011 to 2021, focusing on PC cases.

All cases were retrieved from a Microsoft Access Database (Version 2001, Microsoft Corp, Redmond, WA, US), where patients are registered after discharge. This database contains the following information for each patient: sex, age at surgery, clinical presentation, preoperative calcium, parathormone (PTH), vitamin D 25-OH, phosphorus, creatinine, calciuria, preoperative instrumental examinations, description of surgical intervention, intraoperative PTH, pathological description, weight and diameter of the excised glands, postoperative complications, and follow-up.

All patients underwent both neck ultrasound and 99Tc-labeled sestamibi scintigraphy (MIBI) before surgery. Further imaging was performed only in selected cases: patients referred to us for persistent PHPT after previous neck interventions or suspicion of PC with local invasion. No patient underwent fine needle aspiration (FNA). Calcium is expressed in mg/dL (normal range 8.4–10.2 mg/dL), PTH in pg/mL (normal range 8.7–79.6 pg/mL), phosphate in mg/dL (range 2.5–4.5 mg/dL), vitamin D 25-OH in ng/mL (normal range 30–100 ng/mL), creatinine in mg/dL (range 0.84–1.21), and 24-hour calciuria in mg/kg/24 h (normal value < 4 mg/kg/24 h).

The same experienced endocrine surgeon performed all surgical procedures, and surgical samples—both intraoperative and definitive—were analysed every time by the same pathologist, who has in-depth expertise in parathyroid pathologies. The PC diagnosis was based on (i) the presence of invasive growth involving adjacent structures, such as thyroid and soft tissue, (ii) capsular and/or extracapsular blood vessels or perineural spaces, (iii) and/or documented metastases, based on 2017 WHO criteria.

All patients were classified into two different risk classes, according to Schulte: low risk (capsular and adjacent soft tissue invasion) and high risk (vascular and vital organ invasion).

Postoperative hypocalcemia was considered as a serum calcium value lower than 8.4 mg/dL, with normal PTH. Postoperative hypoparathyroidism was considered serum PTH lower than 8.7 pg/mL and with a calcium value lower than 8.4 mg/dL. Both complications were deemed transient if lasting less than six months and definitive if lasting longer. Hungry Bone Syndrome (HBS) was defined as the need for oral calcium supplementation, with normal PTH levels, due to bone resorption.

All patients underwent postoperative laryngoscopy. Inferior Laryngeal Nerve Palsy (ILNP) was defined as transient or definitive, depending on whether it persisted for less or more than six months, respectively. All patients were monitored by an expert endocrine oncologist, with blood and instrumental tests every three months in the first year and every six months afterwards. We considered patients with adequate PTH and calcium serum levels to be cured.

First, we summarised data and outcomes descriptively for every patient affected with PC.

Then, based on the cause of PHPT, we split the patients into four groups: PC, typical adenoma, atypical adenoma, and hyperplasia.

The data that support the findings of this study are available from a Microsoft Access Database (Version 2001, Microsoft Corp, Redmond, WA, US), upon request to the corresponding author.

### 2.1. Statistical Analysis

We performed a statistical analysis on the data collected to compare patients' demographics, preoperative evaluation results, intraoperative findings, and outcomes among the four study groups.

The Chi-square test was used to assess differences in binomial variables in the four groups. The binomial variables compared were sex distribution, preoperative imaging concordance, overall complication rate, postoperative hypoparathyroidism rate, postoperative haemorrhage rate, recurrent laryngeal nerve lesion rate, and hungry bone syndrome rate.

The Kruskal–Wallis test was used to assess the differences in all other parameters in the four groups. The parameters evaluated via the Kruskal–Wallis test were age at surgery, preoperative blood calcium, preoperative blood phosphate, preoperative blood PTH, preoperative blood creatinine, greatest excised gland weight and diameter, postoperative blood calcium on postoperative days 1 and 2 (POD 1 and 2), and postoperative blood PTH.

All statistical analyses were performed using the SPSS software (PASW Statistics for Windows, version 21.0; SPSS Inc., Chicago, IL). Values of *p* < 0.05 were deemed to be statistically significant.

## 3. Results

Four hundred sixty-two patients underwent surgery for parathyroid diseases: 419 (90.7%) for primary, 39 (8.4%) for secondary, and 4 (0.9%) for tertiary hyperparathyroidism. We excluded patients with secondary and tertiary HPT. Out of the 419 cases operated on for PHPT, 8 (1.9%) were affected with PC and 411 (98.1%) with benign parathyroid diseases: 330 (78.8%) had a histological diagnosis of adenoma (A), 55 (13.1%) of hyperplasia (H), and 26 (6.2%) of atypical adenoma (AA) (Table 1).

### 3.1. Clinical Presentation of PC

The eight patients affected with PC were five males and three females, with a mean age of 57.8 years (range 28–78 years). They all had a preoperative diagnosis of PHPT via blood tests.

  Patient 1: a 38 y.o. male with a long history of kidney stones
  Patient 2: a 73 y.o. male admitted to the Emergency Department for mental confusion with evidence of severe, life-threatening hypercalcemia of 18.0 mg/dL and acute renal failure; he was already known for persistent PHPT after two neck explorations at another hospital
  Patient 3: a 74 y.o. female affected with severe osteoporosis
  Patient 4: a 50 y.o. female with a neck mass initially interpreted as a thyroid nodule
  Patient 5: a 45 y.o. female with leg muscle weakness and tibial and peroneal lesions with suspicious metastasis
  Patient 6: a 78 y.o. male admitted to the Emergency Department with mental confusion and evidence of severe life-threatening hypercalcemia of 19.3 mg/dL and acute renal failure
  Patient 7: a 76 y.o. male with depression and cognitive decline
  Patient 8: a 28 y.o. male with a long history of kidney stones, previous resection of right-hand sarcoma, and partial kidney resection for carcinoma

Median preoperative levels were calcium 13,7 mg/dL (range 12,5–19,3), PTH 736,9 (205.0–4349.0), and phosphorus 2,1 ± 0,425 (0,6–3,3).

A neck ultrasound and a MIBI scintigraphy were performed in all eight cases, which showed evidence of a single enlarged parathyroid gland. In all cases, the two types of imaging confirmed that the lesion was on the same side. Three patients (patients 2, 5, and 6) underwent a CT scan for suspicious PC with adjacent organ invasion—not confirmed in 2 cases—while in 1 (patient 6), the CT scan indicated suspicious oesophagus infiltration. A transoesophageal ultrasound endoscopy ruled out this suspicion. The mean preoperative diameter of the lesion from the neck ultrasound was 25,8 mm (range 15,0–36,0).

### 3.2. Treatment

All eight patients underwent surgery: 2 mini-invasive video-assisted inferior left parathyroidectomies (MIVAP) (patients 1 and 3); 1 MIVAP converted to traditional neck exploration with the removal of three parathyroid glands for intraoperative detection of multiple gland disease (patient 7).

In 5 cases (patients 2, 4, 5, 6, and 8), an enlarged right inferior parathyroid gland was excised en bloc with the ipsilateral thyroid lobe and the superior parathyroid. In these cases, a strong suspicion for PC arose preoperatively based on the excessively high serum calcium and PTH levels, coupled with the large US diameter of the affected gland. In this group of 5 patients, 3 underwent central node dissection and 2 contralateral thyroid lobe excisions: patient 4 had multinodular goitre, and patient 8 had a histological diagnosis of clinically unsuspected papillary carcinoma of the resected right lobe and radicalisation was advised.

In all cases, intraoperative PTH (IOPTH) dosage was performed, considering significant drop of 50% in PTH value ten minutes after parathyroid excision compared to the preincision value.

In patient 4, who underwent total thyroidectomy as part of the same procedure, IOPTH was measured 10 minutes after en bloc resection of the right side and before commencing surgery on the left side.

Among three patients with no preoperative suspicion of PC, patient 1 underwent reintervention after pathological diagnosis. It consisted of ipsilateral lobectomy and VI-level lymph node removal 45 days from the initial intervention. The other two patients (patients 3 and 7) were not reoperated, one for refusal (patient 3) and the other (patient 7) for comorbidities (Table 2).

### 3.3. Outcomes

In all cases, the tumour was removed entirely without capsular rupture, and there was no evidence of resection margin invasion.

In 7 cases, we observed a significant IOPTH drop, with a mean drop of 92.3% (range 86.8–99.1%). In 1 case (patient 7), IOPTH did not decrease after MIVAP excision of the enlarged inferior left parathyroid, based on preoperative localisation. Traditional neck exploration showed significantly enlarged inferior right and minimally enlarged superior left parathyroids, not described by the preoperative ultrasound.

In 5 cases, the PTH value was lower than normal on the first postoperative day (cases 1, 3, 4, 6, and 7). Of these patients, 1 underwent total thyroidectomy (case 4) and 1 subtotal parathyroidectomy (case 7). The other 3 patients showed normal PTH levels on day one (patients 2, 5, and 8), with a mean value of 15.3 pg/mL (range 3.4–42.8).

In 4 cases, first postoperative day calcium values were higher than normal (patients 2, 5, 6, and 8), while they were within the range in the other 4 cases (patients 1, 3, 4, and 7), with a mean value of 10.1 mg/dL (range 8.1–12). In all cases, calcaemia reached the lowest value on the third postoperative day, with a mean value of 8.4 mg/dL (range 7.6–9.2).

Seven cases developed transient hypocalcemia (patients 2, 3, 4, 5, 6, 7, and 8)—4 with associated transient hypoparathyroidism (patients 3, 4, 6, and 7), which became definitive in 1 case (patient 7). Three cases developed hungry bone syndrome (patients 5, 6, and 8).

One transient ILNP (patient 6) and one definitive ILNP (case 5) were observed, the latter one due to intraoperative findings of nerve involvement, which was not suspected preoperatively because the preoperative laryngoscopy showed normal motility of the vocal cords (Table 3).

Pathological examination showed capsular and soft tissue invasion in 3 cases (patients 2, 7, and 8), only capsular invasion in 4 (patients 1, 3, 4, and 6), and capsular, soft tissue, and vascular invasion in 1 (patient 5). 5 patients presented mitosis (patients 1, 2, 3, 4, and 8), with mean mitosis/HPF 3 (range 1–5). Ki67 label index was present in 7 patients (cases 1, 2, 4, 5, 6, 7, and 8) and was higher in the two younger patients (cases 1 and 8). All had fibrous bands.

Patient 7, who underwent the removal of three glands, was diagnosed with carcinoma of the inferior right parathyroid (diameter 1.3 cm, weight 2,930 mg) and hyperplasia of the inferior left parathyroid (diameter 2.5 cm, weight 1,630) and the superior left parathyroid (diameter 0.6 cm, weight 30 mg).

Parathyroid weight could only be ascertained for 3 patients (cases 1, 3, and 7) because the thyroid lobe and the parathyroid carcinoma were weighed together in the five en bloc resections. Median weight was 2,415 mg (range 1,850.0–2,930.0). Median histological diameter was 2,3 cm (range 1,3–3,6).

In all cases, histological examination confirmed that the tumour did not involve resection margins. All cases were metastasis-free. The patient with tibia and fibula lesions had a diagnosis of “brown tumours.”

Patient 8 underwent genetic tests because of suspicious HPT/JT syndrome due to previous sarcoma and kidney carcinoma: CDC73, AIP, CDKN1B, GNAS, HRAS, MEN1, NF1, VHL, MAX, GPR101, PRKAR1a, RET, SDHA, SDHAF2, SDHB, SDHC, SDHD, TMEM127, EPAS1. All tests were negative for mutations.

Based on the Schulte classification, only one patient was high risk, presenting vascular and recurrent laryngeal nerve invasion: pT4N0M0 (patient 5). All other patients were low risk, presenting only capsular and soft tissue invasion: 3 pT1NxM0 (patients 1, 3, and 7), 1 pT1N0M0 (patient 4), two pT2N0M0 (patients 6 and 8), and 1 pT2NxM0 (patient 2).

At a mean follow-up of 38.4 months (range 13–109), all patients were alive and had no recurrence evidence. All patients had normal calcium and PTH levels, with a mean value of 9.3 mg/dL (range 8.5–10.4) and 34.7 pg/mL (range 6.3–88), respectively, and negative neck ultrasound (Table 4).

### 3.4. Comparison between PC and Other Causes of PHPT

Compared with other PHPT patients, PC patients were more frequently male, had higher preoperative blood calcium and PTH and lower phosphate, had larger and heavier parathyroids excised, had lower postoperative calcium levels, and showed a higher rate of postoperative hypoparathyroidism (Table 5).

The Chi-square test showed statistically significant differences between the 4 groups in terms of sex distribution (*p* 0.012), overall complication rate (*p* 0.007), and postoperative hypoparathyroidism rate (*p* < 0.001). There was no statistically significant difference in terms of concordant preoperative imaging rate (*p* 0.168), postoperative haemorrhage rate (*p* 0.573), recurrent laryngeal nerve lesion rate (*p* 0.48), and hungry bone syndrome rate (*p* 0.671).

The Kruskal–Wallis test showed statistically significant differences in terms of preoperative blood calcium (*p* < 0.001), preoperative blood phosphate (*p* 0.015), preoperative blood PTH (*p* < 0.001), greatest excised gland weight and diameter (respectively, *p* < 0.001 and 0.007), and postoperative blood calcium on POD 1 (*p* 0.033). There was no statistically significant difference in terms of age at surgery (*p* 0.429), preoperative blood creatinine (*p* 0.313), postoperative blood calcium on POD 2 (*p* 0.894), and postoperative blood PTH (*p* 0.128) (Table 5).

## 4. Discussion

PC is a rare carcinoma and an infrequent cause of PHPT. The incidence of PC as a primary cause of PHPT is around 1%, ranging from 0.2 to 5.0% in the literature [10–16], 1.9% in our series.

Typically, patients affected with PC present with symptoms and complications of PHPT, such as bone and kidney disease, depression, anxiety, weakness, and gastroenteric symptoms (abdominal pain, nausea, vomiting, pancreatitis, and peptic ulcer). At presentation, 50% of patients show renal and bone manifestations, with different degrees of severity, osteopenia, osteoporosis, osteofibrosis, osteitis fibrosa cystica, and pathologic fractures [1, 10, 17, 18]. Some may present with hypercalcemic crisis [19, 20], 2 in our cohort.

About 10% of cases of PC are not functioning [6–8]. Because of the absence of PTH secretion, they usually present at a more advanced stage, with symptoms of local and adjacent structure invasion [7, 8, 21–23], hoarseness, and/or dyspnea and/or neck mass.

Completely asymptomatic PC has been described [11, 24–26]. In our cohort, seven patients presented with symptoms, which led to PHPT diagnosis, and 1 with a neck mass.

According to other studies, preoperative calcium and PTH values were significantly higher, while phosphate levels were lower than in benign PHPT [3, 5, 24, 27–29]. However, the criteria to define a threshold of malignancy is still under debate.

In our cohort imaging, ultrasound scans and MIBI scintigraphy proved useful in localising the lesion, in that they always identified the same diseased gland, but they did not distinguish between benign and malignant lesions. Only in one case was there the suspicion of extracapsular extension of parathyroid lesion, which CT scan confirmed. In such situations, characterised by an invasion of the surrounding structures, ultrasound scans may anticipate malignancy, as well as in cases of enlarged lymph nodes and/or very enlarged parathyroids: a diameter >3 cm has been reported as suspicious for PC [30–32]. CT and MRI may be useful in PC with the invasion of surrounding tissue and adjacent organs or distant metastases [32, 33].

We had never performed fine needle aspiration on parathyroids, especially when we suspected PC. Fine needle aspiration on parathyroid glands should not be performed preoperatively to prevent capsular rupture and tumour dissemination along the needle tract and the related risk of recurrence [33–36].

Considering the above, the challenge for clinicians is to differentiate between PC-induced PHPT and PHPT due to benign diseases. This issue is the key to guaranteeing the best course of treatment for patients: complete tumour resection with microscopically negative margins and intact tumour capsule, at first intervention, is the best chance of cure.

En bloc resection is the gold-standard treatment. It consists of removing the parathyroid tumour, the surrounding soft tissue, the ipsilateral thyroid lobe, the VI-level lymph nodes, and the adjacent structures (if involved by the carcinoma), avoiding the spillage of tumour cells into the surgical field [33, 37, 38]. En bloc resection seems to reduce the risk of recurrence, compared to simple excision of the diseased parathyroid (8% for the former against 51% for the latter) [39].

However, as PC diagnosis often takes place after surgery, local excision of the affected gland along the border of the peritumoral capsule is the most often performed first-line surgical procedure [40–44]. According to some authors, the prognosis of these patients may improve in terms of local recurrence, with additional surgery consisting of en bloc resection of the ipsilateral thyroid lobe and the central compartment lymph nodes within one month [33, 38, 41, 45]. However, the benefits of reintervention are not clear for patients with local extracapsular excision, and other authors suggest close follow-up for them [21, 44, 46].

There are conflicting views regarding the extent of the intervention as an important predictor of recurrence and death. In some studies, local excision of PC showed to be an adverse prognostic factor compared with more extensive resections [9, 41, 47]. According to other authors, there is no link between radical excision and improved survival [21, 44].

In our cohort, based on the clinical presentation of PHPT, particularly with very high preoperative levels of calcium and PTH and large gland size at imaging, we suspected PC in 5 out of 8 cases (62.5%). This diagnosis led us to performing en bloc resection in all these cases, and pathology confirmed the diagnosis. In the remaining 3 cases, no preoperative elements led us to suspect PC, and simple local excision was the choice. There was no suspicion of malignancy during the intervention in any of these 3 cases since the pathological glands were easily cleaved from the surrounding tissue and were smaller than 2 cm.

In addition to preoperative features, the intraoperative presentation may lead to suspecting malignancy, first, the diameter of the lesion. Many authors cite a median maximum PC diameter ranging from 3.0 and 3.5 cm to be larger than that of simple parathyroid adenoma [1, 14, 48, 49]: our cohort confirms this statement.

PC usually consists of lobulate, solid, hard-consistence tumours, with a cystic component in 21% of cases [33, 45], a dense fibrous capsule, ranging in colour from greyish to white, while adenoma is soft, smaller, and reddish-brown [45, 49]. Adhesions to surrounding tissue and/or invasion of adjacent organs are signs of malignancy [49–52].

The definitive diagnosis, however, is only possible with a histological examination after surgical excision. Schantz and Castleman first reported pathological criteria to define parathyroid carcinoma in 1973: fibrous bands, capsular invasion, vascular invasion, and mitotic activity [53–56].

According to the WHO classification, revised in 2017 [57–59], the diagnosis of malignancy should be restricted to those tumours showing evidence of invasive growth and involving adjacent structures, such as thyroid and soft tissue, capsular and/or extracapsular blood vessels, or perineural spaces and those with documented metastases. Vascular invasion occurs when a tumour invades capsular vessels or vessels of the surrounding soft tissues. Tumour's cellularity is variable: broad bands of fibrous connective tissue extending from the peritumoral capsule often divide cell clusters. These bands are present in 90% and mitotic figures in 80% of PC cases, but both characteristics are not specific for malignancy [59], even if atypical mitoses strongly favour PC diagnosis.

The Ki67 proliferation index is the most studied marker: it is higher in carcinomas (6–8%) than in adenomas (<4%), and a percentage greater than 5% generally suggests PC [60–63]. CDC73, also called parafibromin, is another marker: many parathyroid carcinomas are negative [64]. Conclusions from the literature on genetic profiling require cautious interpretation because studies can differ in the criteria used for diagnosis and selecting cases.

Among the cases selected using rigorous criteria, inactivation of the tumour suppressor gene CDC73 is the major known molecular driver in the pathogenesis of PC. Somatic mutation of CDC73 is present in 70% of PCs [65] and rarely in benign sporadic adenomas (0.8%) [66–69]. Germline mutations are present in one-third of patients [70], suggesting HPT-JT syndrome in a subgroup of patients and occasionally in FIHP [23, 67, 68, 71–80]. The presence of CDC73 mutation with negative parafibromin increases the probability of malignancy [81–90], but it is not pathognomonic. Other genes or their protein products, such as BRCA2, Rb, *p*53, and PRAD 1 [7, 49, 91–94], have been studied in PC pathogenesis, but none has proven useful in distinguishing PCs from benign adenomas.

All patients showed unequivocally WHO histological malignancy criteria in our series, and only one showed vascular invasion. In addition, all had fibrosis bands, 5 had mitosis, and 2 had a very high Ki67 index. Only in 1 case was the CDC73 mutation studied, but it was negative.

There is no universally recognised staging system for PC [95]. A large retrospective cohort study proposed a former staging system [9, 14], which proved insufficient to achieve meaningful outcome prediction in terms of prognosis.

Talat and Schulte proposed a staging system based on histopathological criteria. It includes two risk classes: low risk (capsular and adjacent soft tissue invasion) and high risk (vascular and vital organ invasion). This system seems to have a great power to predict survival and recurrence [9, 95]: patients categorised as high risk carried a higher risk of recurrence and death than those in the low-risk category. According to the Schulte classification, only one patient in our series met the high-risk criteria. All three patients who underwent local excision were low risk. For this reason, we radicalised only the younger patient because one refused reintervention, and the other was an elderly subject with comorbidities.

Our experience, albeit limited to a small number of cases, seems to confirm the validity of the Schulte staging system concerning prognosis. The positive outcomes of our cohort can be explained mainly by the fact that 7 of 8 patients affected with PC were low risk. Furthermore, most of them (5/8), including the high-risk patient, underwent en bloc resection and one radicalisation after the first procedure. This supports our previous arguments about the value of surgery extension as a prognostic factor. Finally, the high-risk patient had a relatively short follow-up period of 16 months.

According to other studies [7, 8, 96, 97], patients affected with PC relapsed within 2 to 5 years from the initial intervention, usually presenting increasing serum and PTH calcium. Distant metastases occur in about 25% of patients during follow-up [27, 98], with a disease-free period of up to 20 years [82, 98, 99]. The most reported metastasis sites are lungs (40%), liver (10%), and, in a few cases, bones, pleura, and pancreas [45, 100]. The clinical course is usually indolent, and some patients survive for many years after diagnosis of metastatic disease.

The main clinical manifestation of recurrence is hypercalcemia and related complications [45]. In some cases, the metastatic disease can detect a misunderstood PC diagnosis in a patient who previously had been operated on for PHPT [25]. In about two-thirds of cases, the main site of recurrence is local in the neck [27] and is difficult to detect because it is small and/or multifocal and/or located in the scar left by the previous surgery. Half of these patients also have distant metastasis [98].

The best treatment, if possible, is reintervention [1, 24, 48, 97, 101–103], with an increase in long-term survival of up to 30% [97, 101]. Even if reintervention is not radical, it can be useful for palliation by reducing serum calcium levels for a period ranging from months to years [1, 12, 24, 27, 82, 99, 101, 102]. In local recurrences, percutaneous US-guided alcohol injections have been reported in the literature, but with short-term improvements in calcium serum levels [104] and RLN injuries associated with ethanol toxicity [105]. For liver and lung metastasis, radiofrequency ablation (alone or with arterial embolisation) has been reported as having a good chance to improve calcium and PTH levels [106, 107]. Therapies other than surgery, such as chemotherapy and radiotherapy, both as alternative and adjuvant therapies, have shown poor results [24, 96]. Few studies report on novel therapies, such as anti-PTH immunotherapies and biological agents [108, 109].

The lack of a universally recognised staging system for PC does not allow clinicians to formulate a precise prognosis. Studies report overall survival rates between 76 and 85% and 49 and 77% at five and ten years, respectively [1, 14, 21, 25, 96]. In patients treated with complete tumour resection during the initial surgical procedure, survival rates improve to 90% and 67% at five and ten years, respectively [110].

In our series, all patients are alive and disease-free, but only two among these have a follow-up period longer than five years: this is a limit of the present study.

The prognosis of nonfunctional PC is worse since diagnosis is always made at an advanced stage [28, 111].

## 5. Conclusions

Although limited by a short follow-up and the small size, our study highlights some valuable aspects to suspect PC and differentiate PHPT-PC from PHPT due to benign causes preoperatively. Suspecting this rare cancer is essential to offer patients the best treatment options at the time of diagnosis.

According to Schulte classification, our series seems to support both the value of surgery extension at first intervention and risk class as prognostic factors for recurrence in patients affected with PC.

The rarity of this tumour and the ability to recognise it among other causes of PHPT requires in-depth expertise. The implicit consequence is that preferably the referral centres should treat these patients.

Due to the rarity of this cancer, there is the need for multicentre studies collating cases from referral centres.

## Figures and Tables

**Table 1:** Causes of PHPT in our series.

Benign				Uncertain		Malignant		Total	
Adenoma		Hyperplasia		Atypical adenoma		Carcinoma			
330	78.8%	55	13.1%	26	6.2%	8	1.9%	419	100%

**Table 2:** Clinical data and treatment of 8 patients affected with PC.

No.	Sex	Age	Clinical presentation	Preoperative calcium	Preoperative PTH	Preoperative localisation	Ultrasound diameter (mm)	Sestamibi scan	Suspicion of PC	Operation	IOPTH decrease (%)
1	M	38	Kidney stones	12.5	289.0	Yes	15.0	+	No	MIVAP	94.8
2	M	73	Mental confusion—acute renal failure	18.0	2160.0	Yes	36.0	+	Yes	En bloc resection	99.1
3	F	74	Osteoporosis	12.7	391.1	Yes	14.0	+	No	MIVAP	86.8
4	F	50	Neck mass	13.2	1055.0	Yes	28.0	+	Yes	En bloc resection	90.7
5	F	45	Tibial and peroneal lesions suspected of metastasis	13.7	4349.0	Yes	30.0	+	Yes	En bloc resection	92.0
6	M	78	Mental confusion—acute renal failure	19.3	2146.0	Yes	31	+	Yes	En bloc resection	91.0
7	M	76	Depression—cognitive decline	12.5	205	Yes	Not found	−	No	Subtotal parathyroidectomy	—
8	M	28	Kidney stones	13.9	772	Yes	23	+	Yes	En bloc resection	92.3

**Table 3:** Postoperative complications of 8 patients affected with PC.

No.	Transient hypoparathyroidism	Definitive hypoparathyroidism	Transient hypocalcemia	Definitive hypocalcemia	Hungry bone syndrome	Transient recurrent laryngeal nerve palsy	Definitive recurrent laryngeal nerve palsy
1	Yes	No	No	No	No	No	No
2	No	No	Yes	No	No	No	No
3	Yes	No	Yes	No	No	No	No
4	Yes	No	Yes	No	No	No	No
5	No	No	Yes	No	Yes	Yes	Yes
6	Yes	No	Yes	No	Yes	Yes	No
7	Yes	Yes	Yes	Yes	No	No	No
8	No	No	Yes	No	Yes	No	No

**Table 4:** Pathological results, risk class, and outcomes of 8 patients affected with PC.

No.	Histologic diameter (mm)	Weight (mg)	Ki67 (%)	Mithosis/HPF	TNM	Risk	Follow-up (months)	Outcomes	Persistence	Recurrence	Death
1	15	1760	20	4	PT1NXM0	Low	109	Cured	No	No	No
2	36	—	3	2	pT2NxM0	Low	68	Cured	No	No	No
3	18	1850	1	5	pT1NxM0	Low	54	Cured	No	No	No
4	28	—	1	1	pT1N0M0	Low	18	Cured	No	No	No
5	30	—	2	0	pT4N0M0	High	16	Cured	No	No	No
6	32	—	2	0	pT2N0Mo	Low	15	Cured	No	No	No
7	13	2930	2	0	pT1NxM0	Low	14	Cured	No	No	No
8	21	—	30	3	pT2N0M0	Low	13	Cured	No	No	No

**Table 5:** Patients' characteristics according to final histology.

	Hyperplasia		Adenoma		Atypical adenoma		Carcinoma		Whole sample	
Demographics, comorbidities, and preoperative evaluations										
	Male	Female	Male	Female	Male	Female	Male	Female	Male	Female

Sex distribution	18 (33%)	37 (67%)	70 (22%)	246 (78%)	9 (35%)	17 (65%)	5 (63%)	3 (38%)	102 (25%)	303 (75%)

Age (years)	58 ± 16,5 (29–79)		62 ± 16,25 (22–85)		65 ± 26 (20–85)		61,5 ± 32,5 (28–78)		62 ± 17 (20–85)	

Hyperparathyroidism symptoms	14 (25%)	41 (75%)	86 (27%)	230 (73%)	8 (31%)	18 (69%)	2 (25%)	6 (75%)	110 (27,16%)	295 (73%)

Hypertension	0 (0%)	55 (100%)	9 (3%)	307 (97%)	0 (0%)	26 (100%)	0 (0%)	8 (100%)	9 (2,22%)	396 (98%)

Kidney failure	21 (38%)	34 (62%)	100 (32%)	216 (68%)	11 (42%)	15 (58%)	2 (25%)	6 (75%)	134 (33,09%)	271 (67%)

Bone symptoms	13 (24%)	42 (76%)	77 (24%)	239 (76%)	4 (15%)	22 (85%)	1 (13%)	7 (88%)	95 (23,46%)	310 (77%)

Neurological symptoms	0 (0%)	55 (100%)	27 (9%)	289 (91%)	3 (12%)	23 (88%)	2 (25%)	6 (75%)	32 (7,9%)	373 (92%)

Thyroid	6 (11%)	49 (89%)	48 (15%)	268 (85%)	4 (15%)	22 (85%)	2 (25%)	6 (75%)	60 (14,81%)	345 (85%)

Gastrointestinal symptoms	3 (5%)	52 (95%)	23 (7%)	293 (93%)	0 (0%)	26 (100%)	0 (0%)	8 (100%)	26 (6,42%)	379 (94%)

Systemic symptoms	3 (5%)	52(95%)	24 (8%)	292 (92%)	1 (4%)	25 (96%)	0 (0%)	8 (100%)	28 (6,91%)	377 (93%)

Other symptoms	13 (24%)	42 (76%)	45 (14%)	271 (86%)	2 (8%)	24 (92%)	1 (13%)	7 (88%)	61 (15,6%)	344 (85%)

Concordant preoperative imaging	25 (45%)	30 (55%)	184 (58%)	132 (42%)	20 (77%)	6 (23%)	5 (63%)	3 (38%)	234 (58%)	171 (42%)

Preoperative serum Ca (mg)	11 ± 1,135 (9,3–18)		11,3 ± 0,8 (8,9–1213)		12,15 ± 0,975 (10,3–14,8)		13,705 ± 2,5375 (12,5–19,29)		11,3 ± 1,29 (8,9–1213)	
Preoperative serum P (mg)	2,7 ± 0,4 (1,8–3,7)		2,5 ± 0,4225 (0,76–4,5)		2,45 ± 0,675 (1,6–3,9)		2,1 ± 0,425 (0,6–3,3)		2,5 ± 0,5 (0,6–4,5)	

Preoperative serum PTH (mg)	157,15 ± 151,55 (70–2000)		181 ± 156,9 (10–1878)		408,4 ± 283,95 (112–1514)		736,95 ± 1271,25 (205–4349)		183 ± 197 (10–2160)	

Preoperative serum creatinine (mg)	0,8 ± 0,18 (0,59–2,4)		0,8 ± 0,2 (0,42–3,85)		0,8 ± 0,4 (0,59–2,2)		0,9 ± 0,375 (0,6–2,68)		0,9 ± 0,2 (0,42–3,85)	

Intra- and postoperative evaluations										

Greatest excised gland weight (mg)	570 ± 1745 (30–30000)		800 ± 1062,38 (6–32000)		1700 ± 2126 (60–9000)		2415 ± 257,5 (1760–2930)		845 ± 1072,38 (6–32000)	

Greatest excised gland diameter (cm)	2 ± 0 (1,2–27)		1,5 ± 0,2 (0,008–5,5)		2,3 ± 1 (0,7–5)		2,05 ± 0,775 (1,5–3,6)		1,7 ± 0,2 (0,008–27)	

Intraoperative serum PTH (mg)	177 ± 117,7 (48–2733)		175,5 ± 116,6 (2–1550)		433,6 ± 569,85 (107,3–1486)		759,75 ± 701,925 (250–2257)		190 ± 144 (2–2733)	

One-day postoperative serum PTH (mg)	72,6 ± 55 (9–640,6)		36 ± 27,5 (2–350)		38 ± 62,85 (11,1–151)		69,5 ± 116,85 (16,6–250)		38 ± 32,6775 (2–640,6)	

One-day postoperative serum Ca (mg)	9,185 ± 1,5 (7,4–12,7)		9,1 ± 0,9675 (6,79–13,3)		9,4 ± 1,075 (8,3–11,5)		10,25 ± 0,975 (8,1–12)		9,17 ± 1 (6,79–13,3)	

Two-day postoperative serum Ca (mg)	8,74 ± 1,02 (7–11,4)		8,7 ± 0,625 (6,98–13,9)		8,55 ± 0,775 (7,5–10)		8,95 ± 0,55 (7,6–9,7)		8,7 ± 0,71 (6,98–13,9)	

Postoperative serum PTH (mg)	18,85 ± 24,765 (3,6–400)		20,2 ± 20,09 (1–581)		16,3 ± 16,05 (5,9–75,1)		10 ± 11,075 (3,4–42,8)		19,2 ± 21,2 (1–581)	

Complications and recurrences										

	Yes	No	Yes	No	Yes	No	Yes	No	Yes	No

Surgical complications	18 (33%)	37 (67%)	81 (26%)	235 (74%)	12 (46%)	14 (54%)	5 (63%)	3 (38%)	116 (29%)	289 (71%)

Transient hypoparathyroidism	8 (15%)	47 (85%)	26 (8%)	290 (92%)	6 (23%)	22 (85%)	4 (50%)	4 (50%)	44 (11%)	361 (89%)

Definitive hypoparathyroidism	2 (4%)	53 (96%)	1 (0%)	315 (100%)	0 (0%)	26 (100%)	0 (0%)	8 (100%)	3 (1%)	402 (99%)

Haemorrhage	0 (0%)	55 (100%)	8 (3%)	308 (97%)	1 (4%)	25 (96%)	0 (0%)	8 (100%)	9 (2%)	396 (98%)

Transient recurrent laryngeal nerve lesions	1 (2%)	54 (98%)	9 (3%)	307 (97%)	1 (4%)	25 (96%)	0 (0%)	8 (100%)	11 (3%)	394 (97%)

Definitive recurrent laryngeal nerve lesions	2 (4%)	53 (96%)	6 (2%)	310 (98%)	0 (0%)	26 (100%)	1 (13%)	7 (88%)	9 (2%)	396 (98%)

Hungry bone syndrome	2 (4%)	53 (96%)	21 (7%)	295 (93%)	2 (8%)	24 (92%)	1 (13%)	7 (88%)	26 (6%)	379 (94%)

Hyperparathyroidism persistence	6 (11%)	49 (89%)	3 (1%)	313 (99%)	0 (0%)	26 (100%)	0 (0%)	8 (100%)	9 (2%)	396 (98%)

Hyperparathyroidism recurrence	1 (2%)	54 (98%)	3 (1%)	313 (99%)	0 (0%)	26 (100%)	0 (0%)	8 (100%)	4 (1%)	401 (99%)

## Data Availability

The data that support the findings of this study are available from a Microsoft Access Database (version 2001, Microsoft Corp, Redmond, WA, US) upon request to the corresponding author.
